# Materia Medica and Therapeutics

**Published:** 1835

**Authors:** 


### REVIEWS AND BIBLIOGRAPHICAL NOTICES.

---

### Art. VII.—Materia Medica.

*Materia Medica and Therapeutics, by Dr. A. G. Thompson. Second Edition,* 1 *vol. pp.* 10–73.

(From a Correspondent.)

Professor Thompson, of the London University, is a man of extensive acquisitions in medical science. The work, an analysis of which we are about to present to the readers of this journal, is replete with instructive matter. Were our object a critical examination of the work, we could, like all other wise critical detectors of defects, discover much in its pages upon which our eager appetite for fault-finding might feed. There are errors, and serious ones, in this book, and were it not for its numerous excellencies, these errors would drag it down to rapid oblivion. But the inherent merits of the work will not only keep it from passing to the tomb of forgetfulness, but will urge its onward way to celebrity, and to a high appreciation of its numerous excellencies.

"The intention of the following pages," says Dr. Thompson in his preface to the first edition, "is to present a condensed view of the branch of medical science of which they treat. The extent and nature of the subject has been too little considered, and the preliminary acquisitions requisite for its proper acquirement most unaccountably overlooked in the course of studies prescribed by the incorporated medical bodies in this country." The preliminary acquisitions here referred to by Dr. Thompson, are those of natural history, botany, chemistry, anatomy, and physiology, the pretermission of the study of which by the student of medicine is a just subject of reprehension. Yet there is another branch of preliminary study which should never be omitted by the student of our science —that of intellectual philosophy. And by intellectual philosophy we mean neither the erring fancies and flights of phrenology, nor the deceptious speculations of metaphysical subtlety, but a sound, authentic, enlightened view of the capacities of the mind, and of their appropriate methods of exercise in search of truth. There is a spirit or taste of the mind, sometimes the gift of nature, but oftener the result of a protracted discipline of its powers, which is far more valuable than all the limited acquirements of our youthful days; without which the noblest capabilities of genius are often lavished on topics of inquiry in themselves transcendental, or of very inferior consequence in science, but which a fond fancy clothes in the drapery of exaggerated importance. The history of materia medica bears emphatic testimony to the truth of the above remark, for during many ages it was little else than a history of vain attempts to discover universal specifics, and to substitute mere a priori reasoning for well ascertained facts. Even in this philosophic age of careful examination, of pains-taking experiment, of logical deduction, many errors are warmly cherished by physicians, on the virtues of medicinal substances. The laws of the animal economy are often lost sight of, in our speculations on the agency of remedies, and the materia medica seems reared up as a distinct branch of practical medicine, on ground alien from the territory of a just conception of the properties of life, and of their modes of action in health and disease.

But to proceed with our author. "Although the greatest portion of the work can be regarded only as a compilation, yet the author has introduced into its pages the results of 30 years of attentive and close observation in the treatment of diseases. With an enthusiastic love and a veneration for his profession, which he is anxious to impress on those who are commencing the study of its principles, he has endeavored to trace the nature of the phenomena of morbid action, and to ascertain the actual influence exerted by remedial agents in effecting its removal." We have copied the above declarations of the author, because they reveal a proper and noble generosity in the mind of the writer, which should preposses us in his favor.

Dr. T. commences his work by a consideration of the general action of medicines, he then proceeds to a classification of medicinal agents, after which he discusses the properties of these agents, under their respective divisions of excitants, sedatives, refrigerants, etc., and closes by an appendix, containing tables of chemical equivalents, or atomic weights of substances belonging to the materia medica, and of ultimate components of several articles of the materia medica.

In part 1, section 1, we have some good reflections on the "general circumstances connected with the action of medical agents on the living body." "Medicines of an animal and a vegetable nature differ from food, in containing some active principle not adapted to repair the waste of the body; which resists the digestive powers, and often governs them: their other components, namely, resinous, albuminous, gelatinous, saccharine, oleaginous, feculaceous, and gummy matters undergo digestion.

"Medicines operate only on the living body: indeed, they may be regarded merely as means of affecting the vitality of the solid, whether that exhibit itself under the phenomena of *contractility*, *irritability*, or *sensibility*, or those that follow volition and association. To produce their effect they must be in immediate contact with some sensitive or irritable part of the body."

Dr. Thompson defines the term "medicine," a substance which is capable of altering the state of the body, whether in health or disease.

He contends that "there are five distinct modes in which medicines act upon the living body: they may act by a direct impression upon the surface to which they are applied, the effect being confined to the part: 2, by an impression upon the nerves of the surface to which they are applied, and the effect to be extended to the other parts of the system; 3, they may be conveyed by absorption, undecomposed, into the system, and influence the habit through the medium of the circulation; 4, they may be decomposed, and operate only by one or more of their constituents; 5, they may operate by counter-irritation, or revulsion; 6, they may exert a chemical action on the tissues."

Medicines, agreeably to our author's views, act locally, when their influence is restricted to the part to which they are applied, as is the case of astringents, when given to check diarrhœa, or in the effect of collyria on an inflamed conjunctiva. The action of medicines on the nervous energy, the effect being general, is abundantly illustrated in the results of medication springing from the introduction of remedial agents into the stomach. "That every medicine which operates on the living solid exerts its influence, either directly or indirectly upon the nervous system, can scarcely be denied. Even when we admit that a medicine, which is absorbed and taken into the circulation, causes chemical changes in the circulating mass, still we must refer its ultimate effects to the nervous system, unless we suppose that the secretions are mere chemical changes in the fluids, altogether independent of the vital principle—an idea which is totally devoid of support." p. 5.

Even when a medicine is absorbed, according to Dr. T.'s views, it must be conveyed to a particular organ on which it is to act, and exert a direct agency on its living solid, which becomes "excited in the same direct manner as the surface to which the medicine was first applied; but at the same time, phenomena occur in distant organs can only be referred to nervous sympathy."

"Again," says he, "upon the whole there is abundant evidence to prove that many medicines produce their effects by not acting directly on the nervous energy independent of absorption. It is not essential that we should be able to demonstrate in what manner this communication with distant parts of the body is effected by the nerves. The attempt to explain the phenomena is as vain as that respecting the vital principle; we find ourselves out of our depth, and the struggle only convinces us that we are in an element which does not belong to us."

There are three surfaces upon which medicinal impressions are made—the gastric and intestinal mucous tissue—the skin—and the organ of smelling. Dr. T. has omitted the mucous surface of the bronchii—an omission which is singularly unwise, for we have the most satisfactory proofs afforded in the mercurialism, induced by the vapors of quicksilver; in the rapid action of the nitrous oxide gas, when the nose is compressed in the act of inspiring it, and in other instances of foreign influences imparting their peculiar mode of modifying the vital properties of the system, through the pulmonary organs, that this channel of nervous susceptibility should never be overlooked in our inquiries respecting the agency of healthful, morbid, and medicinal impressions. It is true that we do not often apply our remedial agents to this point of nervous excitability, for, from the distinctive nature of the organic arrangements which obtain, in the lungs and of the special offices they subserve in the economy, a very limited opportunity is afforded for the administration of remedies through that medium. There are three modes in which a salubrious, or insalubrious atmosphere may affect the lungs. First, there is the essential action of oxygen in operating a change on the blood—secondly, there is the inspiriting power of a pure, free, well ventilated air on the nervous susceptibility of the pulmonary apparatus—and thirdly, there is a certain aerial temperature eminently conducive to their state of functional activity. A deprivation of oxygen leads to immediate death—a diminution of the proper quantity of it for the purposes of respiration, will, of course, induce a corresponding reduction of vital activity. Foreign matters in the atmosphere,—miasmata of various kinds—contagious effluvials affect the animal economy through the pulmonary mucous tissues. The pent up, stifling air, of a large city, although it may not be impregnated with paludal emanations, exerts a deteriorating influence upon the system—as soon as the fresh and free atmosphere of the country is breathed, the lungs dilate with renewed vigor, and the whole system feels refreshed and invigorated. The physical qualities of the air we respire exert a manifest agency on the lungs.

But to return to the text. Dr. Thompson is decidedly of opinion that medicines are absorbed in their entire state, and that this absorption may be effected either by the intestinal tube, by the skin, or through the lungs. Some medicinal substances are however decomposed by the stomach, after which gastric decomposition, they enter the system.

Our author makes some judicious remarks on the revulsive or counter-irritant action of medicines. Medicines operate often in this manner, and change the seat of morbid irritation, setting up a new centre of abnormal perceptions, in which an artificial and transient and manageable disease is substituted for one of a persistive and destructive character. Any potent medication exerted on the skin, alimentary canal, urinary apparatus, or salivary glands, may be effectively remedial in the cure of various morbid affections in organs whose sympathetic and functional acts are in consentient or antagonist relation to the surface, or structure influenced by our revulsions. Thus we blister to drive irritation from the stomach to the skin—we purge to create a revulsion of action from the head to the alimentary tube—we exhibit diuretics to produce a derivative action upon the kidneys, which shall subvert the hydropic effort going on in the capillaries of the subcutaneous cellular texture in anasarca.

"General effects of medicines on the vital solids and fluids, and on the functions;" under this head our author institutes an inquiry into the effects resulting from the action of remedies upon the living solids, and on the fluids, and on the functions of the principal organs.

The effects of medicines on the living solid are either stimulant or sedative—what the living solid implies is not easy to define—the term is employed to express an indefinite idea of what we suppose to be the ultimate fibril of the organic tissues;—every change induced by medicinal substances in the condition of any organ, results from an anterior modification of action wrought in this fibril.

Variations in the circulation may produce some difference in the state of the blood; "but this, if it be the effect of medicines, can be attributed only to their influence on the living solid, not to any chemical union or combination of the medicinal agent or its components with the principles of the blood. The blood may be mingled with medicines which have been taken into the stomach, and have passed into the circulation, either in whole or in part: but, during life, we see no reasons for believing that any material alteration, even of the physical properties of the blood, occurs from this cause. Some late experiments of Dr. Stevens, on the effect of saline substances upon the blood, seem to be at variance with this opinion: but these are too little understood to be brought forward as opposing the opinion which has been advanced." p. 19.

The general effects of medicines on the functions is evinced in the operation of many articles of the materia medica. Digitalis acts on the function of the heart, diminishing the pulsations of that organ; hyoscyamus and other narcotics abate nervous mobility—mercury increases the secretions of bile and saliva.

There is a just distinction which obtains between the physiological and the curative, or therapeutical effects of a medicine. The physiological action of a medicine depends upon the immutable and constant influence of the active principle of the substance operating on the organic tissues;—the therapeutical effects of a medicine are modified by various external and internal contingencies, which attach to the case for which it may be prescribed. Age, sex, idiosyncrasy, climate, and other contingent causes, besides diseases which exert the greatest amount of such modifying agency, these all, in a greater or less degree, exert a disturbing force on the constant regularity of medicinal agency. "Medicines exert no specific influence in curing disease, thence the terms febrifuge, antispasmodic, anti-scorbutic, and such like, are merely conventionals, to announce a secondary result consequent upon the appropriate action of some medicinal agent upon the vital solid." There are "many and very different circumstances which modify the curative operation of medicines." The chief modifying circumstances which exert a force upon the action of remedial agents, are, original conformation of body, manifested in symmetrical peculiarity, or relative proportion as regards the head, extremities, and trunk of the body to one another;—in constitution, in idiosyncrasy, and in temperament; 2, age; 3, sex; 4, custom; 5, climate; 6, mental affections; 7, diseases, its stage, cause, seat, etc., and we might add in addition to these modifying influences, the effects of remedies which have preceded the medicines we administer.

Dr. Thompson, after copying the classifications of remedial articles given by Drs. Young and Murray, and urging objections to both of them, lays down his table of classification, which is the following:

I. Vital Agents.

A.—Influencing the body generally.
a.—By operating directly on the nervous system.

| | | |
|---|---|---|
| *Increasing action: | | Excitants. |
| **Diminishing action: | Primarily, | Sedatives, Refrigerants. |
| | Secondarily, | Narcotics, Antispasmodics. |

b.—On the muscular and sanguiferous systems:

Tonics.
Astringents.

c.—On the secerning system:

Errhines.
Sialagogues.
Expectorants.
Emetics.
Cathartics.
Diuretics.
Emenagogues.
Diaphoretics.

B.—Influencing the body solely by their action on the part to which they are applied:

Epispastics.

a. Rubefacients.
b. Vesicants.
c. Erodents.

II. Chemical Agents.

A.—Influencing the body, or its contents, by their chemical properties:

| | |
|---|---|
| *Acting on the surface: | Escharotics, or Cauterants. |
| **Acting on contents of cavities: | Antacids. Antalkalies. a. Antiseptics. Antilithics. |

III. Mechanical Agents.

Demulcents.
Diluents.

Such is our author's classification, which, though we wish to abstain from criticism, we cannot refrain from the expression of our opinion, as radically erroneous. It is both too redundant, and too meagre. Redundant in the three-fold general divisions of vital, chemical, and mechanical agents—and meagre because he has omitted a very important class of medicines—alterants—from his table. We cannot enter upon this subject now, but will, in some future number of the journal, when we hope to show that a great error, in materia medica writers, has been in excluding alteratives entirely from a distinct consideration on their pages.

Excitants, agreeably to our author's classification, are the first class of medicines treated of. They may be defined—"substances that augment, powerfully, the motions peculiar to the different organs of the body, by a primary impulse on the sensibility and irritability of the part to which they are applied, communicated by the nerves to the whole system." Their general effects consist, 1, in an augmentation of nervous susceptibility—2, an increased action in the moving fibre—3, an acceleration of the frequency, and enhancement in the force of the pulse—4, a higher temperature of the body. There is a distinction between excitants and tonics. "Excitants increase the mobility of the system:—Tonics augment the strength of the muscles: Excitants exhaust the excitability; Tonics, within a certain limit, maintain it; the action of excitants is immediate, powerful, and transitory; that of tonics is slow, almost imperceptible, and progressive, but permanent." We need not give a table of excitants, they are familiar to every student. Dr. T. has placed iodine and mercury among the excitants. This is a bad collocation, for neither of them are stimulants, agreeably to his own definition. They are both alterants, and should have been placed under a distinct division. Strychnia, the active principle of nux vomica, is an excitant, which given in small doses increases the digestive energy, improving the appetite, and augmenting the powers of assimilation to such a degree as to increase the strength and size of the body in a rapid manner. If the dose is large, the respiration becomes oppressed, the respiratory muscles suffer a clonic contraction, and the person feels as if about to be suffocated. The circulation is not sensibly affected, and the urinary organs are little influenced, but the capillary vessels of the skin are powerfully excited, and copious perspirations occur during the action of strychnia. Dogs, cats, and rats, are affected by strychnia, or nux vomica, with symptoms of tetanus, distention of the limbs, tremors, immobility of the eyes, etc. Hogs and goats eat nux vomica with impunity. M. Desportes found that it produces very little effect upon poultry. Tincture of iodine is an antidote to strychnia. Strychnia produces no effect on the system when the spinal marrow has been previously destroyed. It acts, therefore, on the motor set of nerves, the anterior nerves of the spinal column. Strychnia has proved useful in pyrosis, chronic diarrhœa, and dysentery. Dr. Beardsley has found it efficacious in amenorrhœa. The extract of nux vomica, as well as the strychnia, have been of late much employed in paraplegia and other forms of palsy. The dose of strychnia is from one-twelfth to one-fourth of a grain—that of nux vomica half a grain to two grains. Neither of these active articles should be administered where there exists any symptoms indicative of inflammation in the brain in paralytic affections. The author enters into rather an uncalled for explication of the best modes of accumulating and administering electricity for medical purposes. Fourteen pages are thus occupied. Twelve pages are appropriated to caloric, as an excitant—and much of the matter on these twelve pages are irrelevant to the strict points of materia medica.

Iodine is employed as a general excitant, both in its uncombined and its combined state. Simple iodine may be prescribed in the form of pills, or in tincture. M. Luyal prefers the aqueous solution. There are several combinations of iodine with other substances—such as the ioduret of sulphur, ioduret of potassium, (hydriodate of potassa,) ioduret of iron, ioduret of mercury, ioduret of lead, and ioduret of arsenic. As a local application, the ioduret of potash is employed by friction over scrofulous tumors.

Iodine and its preparations stimulate in a special manner the capillary system. As a therapeutical agent, iodine has proved successful in bronchocele, and in scrofulous affections. In that form of scrofula which attacks the mesenteric glands, its employment is of little avail.

Mercury was considered by the ancients as a poison. In its metallic form it exerts no influence on the animal frame; "combined with sulphur, iodine, chlorine, cyanogen, oxygen, and acids, it enters the circulation, exciting powerfully the whole of the glandular system, and increasing in a remarkable degree both the secretions and excretions." We believe in neither of these positions of the author. Mercury, agreeably to our conceptions of its modus agendi, neither enters the blood, nor is it in a strict and accurate sense a stimulant. On these two points we cannot forbear copying a page or two from an essay lately published by us on the subject, entitled "Diseases induced by Mercury."

"There are several very important respects in which, if mercury be a stimulant, it differs from all other articles of that class of remedies. First, other stimulants are withheld by the enlightened practitioner in conducting the treatment of diseases of acknowledged inflammatory character. Take, as examples, dysentery, acute hepatitis, iritis, puerperal peritonitis; to administer brandy, camphor, or any other stimulant, for the cure of these diseases, would be a strong evidence of dementation. And yet mercury is the sheet anchor of the treatment in such diseases. In the second place mercury refuses to act when the system is too much prostrated in its powers. But stimulants are always given when the vital energies are sunk for the purpose of increasing them. * * * * To secure and perpetuate the train of action created by stimulants, we have to increase the quantity administered, and often to change the agent, because the impression induced by them on the organic sensibility is constantly diminishing. It is otherwise with mercurial excitement: to continue the remedy in a less dose than the one commenced with, after the process of mercurialism is instituted, is to greatly enhance the effect. The period which sometimes elapses between the administration of mercury and the development of the salivant effects of the remedy is often considerable. In this a wide difference is seen between its action and that of any agent belonging to the class of stimulants. Swediaur, in his work on Venereal, mentions a case in which the mercurial action did not break forth till more than a month after the use of the mineral! * * * John Hunter, Philip Syng Physick, Bostock, Christison, with many of the other acute and herculean minds of the profession in modern days, have never been able to detect mercury in the blood, saliva, and bones, in the manner *it is said*, the wonder-loving explorers in this dark mine of inquiry, in former ages, found so readily and in such abundance. * * * If mercury enters the blood in one patient, it should do so in every one, to whom it is given to an extent sufficient to salivate. If it does so in every case, why did not Physick, Seyhert, Bostock, Christison, Mayer, Devergie, and many other acute experimental inquirers detect it, assisted, as they were, by the lights of modern chemistry?"

But to return to the work before us. Dr. T. minutely notices the various pharmaceutical preparations of mercury. These are to be found in the common works on materia medica, and in the Dispensatories.

"It is curious to observe," says he, "the extraordinary revolution which has taken place, at various periods, with regard to calomel. The ordinary dose, at present, when we are desirous of bringing the system under the mercurial influence, is from one grain to two grains, combined with opium, taken every night, or every morning or evening, or, at the utmost, three times in twenty-four hours, until the gums be affected. Schrœder relates that, in his time, the dose was half a drachm; Geoffrey makes it from six grains to thirty; Neuterus gave, at first, fifteen grains, for the second dose a scruple, for the third half a drachm, and for the fourth a scruple, which he continued till salivation was induced; and Michaelis Albertus informs us that Helwichius gave five scruples for a dose to two patients, and seventy-two grains to a third, which affected the mouth for a fortnight. Even when it was not intended to affect the mouth, scruple and half drachm doses were very common in the seventeenth century; and at this day in India, scruple doses are usually prescribed in the bilious remittents of that climate." p. 275.

Among many excellent remarks and suggestions in reference to the employment of mercury, Dr. Thompson has made some very superficial and erroneous reflections. Thus, he recommends that calomel should be rubbed on the gums, to produce salivation, and credulously adheres to the old notion, contrary to the best modern lights, that metallic mercury can be found in the bones of persons who have been ptytalized. Syphilis, he contends, is best treated by mild mercurials, pushed to a very gentle salivation.

Under the class of excitants, we have joy and impetuosity —but these remedies are not of easy administration—for "therein must the patient minister unto himself."

The therapeutical employment of excitants is rather rapidly dwelt upon—and in his remarks on that point we see nothing worth transcribing.

"Sedatives—Medicamenta Sedantia.—Sedatives are substances which directly depress the energy of the nervous system, diminishing motion in animal bodies without previous excitement. Regarded in a remedial point of view, they are powers intended to diminish preternaturally increased action in animal bodies. * * * In general, writers on the materia medica have confounded sedatives with narcotics; from which however they are perfectly distinct." Sedatives act on the nerves of a part to which they are applied, by a diminution of their capacity to feel impressions, and upon the irritability of the moving fibre, by lessening its energy. When introduced into the stomach, they induce a sedation of its functional activity, and through the gastric nerves influence the heart, brain, etc.

Sedatives, from the nature of their effects, may be arranged under two distinct heads:—direct sedatives, or those which operate immediately on the nerves; and indirect sedatives, or those which operate through the medium of the vascular system.

The direct sedatives are cyanogen combined with hydrogen, constituting hydrocyanic acid, laurel water, and volatile oil of almonds, or combined with potassium, in cyanide of potassium. Empyreumatic volatile oil,—in tobacco smoke: nicotiana, contained in the leaves of nicotiana *Tabacum*; sulphur, combined with hydrogen, in sulphuretted hydrogen gas, and hydrosulphuret of ammonia: and carbon, combined with hydrogen, in carburetted hydrogen gas.

The indirect sedatives are carbon, combined with oxygen, in carbonic acid gas, and bloodletting.

On the therapeutical employment of hydrocyanic acid and its compounds the author dwells with some particularity. In no kind of idiopathic fever has hydrocyanic acid been employed. In some of the phlegmasiæ it has been found beneficial, especially in phrenitis and pleurisy. As an external application it has proved useful in impetigo; in combination with acetate of lead, in the proportion of f℈ij. of the hydrocyanic acid, sixteen grains of acetate of lead, half a fluid oz. of alcohol, and f℥viij. of *distilled* water. In *prurigo*, *inveterate psoriasis*, and several other cutaneous affection, attended with severe itching and tingling, it has rarely failed in affording relief. In active hemorrhagic affections, experience authorizes the author to place much confidence in this acid. In cases of this character he has augmented the dose from m. iij. to m. xij., or until the pulse gave indications of the power of medicine, and in but few instances, where there was not some serious organic disease, have his expectations been disappointed.

In phthisis its powers have been greatly overrated—it is of less utility as a palliative in this mortal malady than opium. In the last stages of consumption it hastens the final catastrophe, by increasing the patient's debility. It is almost a specific in that affection of the trachea which has been named phthisis trachealis; and which, if the career of inflammatory action in the mucous membrane be not checked, is as fatal as tubercular phthisis itself. In chronic catarrh it is often efficacious. In dysentery it is of great service, given to the extent of four minims, or drops, for a dose, with calomel. Dr. Elliotson has published some cases of dyspepsia, in which the hydrocyanic acid was of signal efficacy. Three drops with ʒi. of the tincture of calumba in cold water, Dr. T. recommends as the best mode of exhibition. In pyrosis and asthma it is of great benefit. In hooping-cough it is to be regarded "as the sheet-anchor of the practitioner;" few cases of this disease, our author thinks, would prove fatal, were the hydro-cyanic acid early resorted to and judiciously administered. "After emptying the stomach with an emetic, and purging briskly, the use of the acid should be begun, and the prescription never altered, except to increase the dose. When thus treated, the disease seldom continues more than a month or five weeks, particularly if the little patients be confined to a graduated temperature, and their diet be solely milk and vegetable." p. 325.

"It has been proposed to employ the essential oil of bitter almonds, instead of the free hydrocyanic acid; on the plea that the strength of the medicine can be more certainly determined; that it is less likely to suffer change from air, light variations of temperature, and age. Twelve drops of the volatile oil of the bitter almond may be regarded as equivalent to m. iv. of the ordinary medicinal acid. One objection, however, to its use, is the powerful effect which it has on individuals of a peculiar idiosyncrasy; namely, that of producing a rash, resembling nettle rash. In some people, eating a single bitter almond will produce nettle rash, vomiting, and vertigo; and it is probable that this depends on something in the fruit, independent of the hydrocyanic acid; as instances are met with in which the sweet almond causes the same inconveniences, nevertheless no hydrocyanic acid has ever been found in it."

"I once ordered it as an ointment, largely diluted with spermaceti ointment; but the effects were such as to prevent me from again prescribing it; and Coullon has recorded the case of a child who was killed by the leaves of the prunus lauro-cerasus applied to a sore on the neck." p. 326.

We will pass over his remarks on the other sedatives till he reaches bloodletting. There are some very important practical observations under that head. "During life the bloodvessels are always in a certain degree of tension, by which the tone of the system is maintained; bloodletting diminishes this tension, and is followed by a relaxation and debility; consequently this operation produced a sedative effect on the frame."

The circumstances which modify the result of bloodletting are—1, the mode of performing the operation, whether by opening a vein or artery; by general or topical methods; by a small or large orifice—2, age—3, temperament—4, sex—5, mode of life—6, climate—7, quantity abstracted; and, we might add, the nature, stage, etc. of the disease. Very young and aged persons bear bloodletting worse than those in the middle periods of life. Men tolerate bleeding better than women—and agricultural people will sustain the loss of blood better than inhabitants of a city. Persons of sanguine temperament, our author thinks, bear bloodletting worse than those of a bilious or phlegmatic temperament. We are of a totally opposite opinion. Venesection is, ceteris paribus, better borne by dwellers in a temperate climate than those of tropical or hyperborean regions.

The quantity of blood drawn has its effects materially modified by the size of the orifice through which it may be detracted. A smaller amount of blood, lost in a rapid manner more deeply affects the circulation, than a larger slowly drained off. Dr. T. controverts Dr. Marshall Hall's position respecting the indispensableness of inducing syncope in cases of severe inflammatory attack. We are not always to wait for syncope, for this is not absolutely essential to secure the beneficial sedative influence of bloodletting. Neither will the rapid superinduction of deliquium animi by the use of the lancet afford any sure evidence that the inflammatory action has been arrested. Cupping, for rapid extraction of blood from a part, is preferable to leeching.

"Practical Employment of Sedatives.—All the sedatives which we have examined, with the exception of bloodletting, are powerful poisons; nevertheless, when they are properly administered, andtheir effects care fully watched, they are possessed of powers that cannot be obtained from any other medicines. They are chiefly indicated in diseases of increased sensibility and irritability. As the remedial effects of most of the substances described have been already mentioned, I will confine my remarks chiefly to bloodletting. In intermittent fevers, sedatives are not indicated, unless we except bloodletting, which was employed as the sole means of cure in these diseases by the older physicians. The practice partly arose from the mistaken notion that a morbific matter existed in the blood, and required to be evacuated—an opinion now justly exploded."

"Whilst the cold or sweating stage is present, it should never be resorted to; for in the first the vital powers are depressed, and in the second they are exhausted." The symptoms during the interval indicating the loss of blood are, fulness and tenseness of pulse, headache, throbbing at the temples, and flushed face.

In synocha, or open inflammatory fever, bloodletting is of great moment, and should be rigorously employed. In large towns fevers require less the use of the lancet than those occurring in the country; in damp situations than in dry; and in crowded hospitals than in private practice.

In typhus, bloodletting employed at the commencement of the disease, will accomplish good results.

In plegmasia, bloodletting constitutes the main curative agent. When, however, the protecting influence of the local inflammation is withdrawn, or greatly abated, then the hand of vascular depletion must be stayed.

In opthalmia, croup, pneumonia, pleurisy, and in the hemorrhages accompanied with general momentum of circulation, the necessity of venesection is obvious.

"In phthisis, bloodletting has been much employed. In the inflammatory stage of the disease, bloodletting is our principal remedial agent. It must be early employed; the object being to diminish vascular action; but in this deplorable malady the symptoms are not always so obvious as to lead to decisive measures; the inflammatory action steals on insidiously, and the disease, when it is only expected, has already established itself in the frame. Still, if suppuration have not taken place, the lancet may prove useful; but the utmost discrimination is required in directing its employment. In the second stage of the complaint, when tubercles are in a suppurating state, much relief has been afforded by small and frequently repeated venesections; the quantity taken at a time should not exceed a few ounces; seldom more than three. Phthisical patients, even after hectic shows itself, bear such bleedings well; and instances are recorded in which they have been bled two hundred times in a few months." p. 350.

In dysentery, contrary to our experience, Dr. T. does not employ the lancet.

"Spasmodic and convulsive diseases depend on increased irritability, and, consequently, whatever favors debility is to be avoided. They are sometimes, however, complicated with an inflammatory diathesis: in which case bloodletting is not to be neglected. When there is a determination to particular parts, although the phlogistic diathesis be not present, cupping and leeches may be proper.

"In apoplexy, the lancet is resorted to during the attack, and also in the interval to prevent the recurrence of the attack, from the idea of congestion or effusion of blood within the cranium: but this is not always the case; and apoplexy may proceed from causes depressing the powers of life, without the presence of actual plethora. When a state of plethora really exists, with an evident determination to the head, bloodletting cannot be dispensed with.

"In mania, the general use of the lancet has produced much mischief. It is true mania is accompanied with the most violent excitement: but in no case is this state so rapidly followed by collapse. How far the employment of other sedatives may be safely proceeded with in these cases is still a question for experience to decide."

"Refrigerants—'Medicamenta Refrigerantia.'—Under this head the author indulges in quite an idle display of speculative ingenuity—which we shall pretermit. His table of refrigerants is as follows:

"Refrigerants operating on the organic functions, are the vegetable acids—nitrate of potash, and biborate of soda:—

those operating on the sensibility are, cool air, ice, cold water and evaporating lotions." The benefits resulting in various diseases from cool air, cold water, ice, and evaporating lotions need no special enumeration at our hands. The use of these measures is so familiar to all engaged in the practice of the profession, that we shall say nothing about them.

J. P. H.

(To be continued.)

